# End-Point Prediction of Granule Moisture in a ConsiGma^TM^-25 Segmented Fluid Bed Dryer

**DOI:** 10.3390/pharmaceutics12050452

**Published:** 2020-05-14

**Authors:** Jakob Rehrl, Stephan Sacher, Martin Horn, Johannes Khinast

**Affiliations:** 1 Research Center Pharmaceutical Engineering GmbH, 8010 Graz, Austria; jakob.rehrl@rcpe.at (J.R.); stephan.sacher@rcpe.at (S.S.); 2 Institute of Automation and Control, Graz University of Technology, 8010 Graz, Austria; martin.horn@tugraz.at; 3 Institute of Process and Particle Engineering, Graz University of Technology, 8010 Graz, Austria

**Keywords:** continuous manufacturing, soft sensor, process modeling, continuous drying

## Abstract

Continuously operated pharmaceutical manufacturing lines often consist of a wet granulation unit operation, followed by a (semi-) continuous dryer. The operating conditions of the dryer are crucial for obtaining a desired final granule moisture. Commercially available dryers lack of a thorough online measurement of granule moisture during the drying process. However, this information could improve the operation of the equipment considerably, yielding a granule moisture close to the desired value (e.g., by drying time and process parameter adjustments in real-time). The paper at hand proposes a process model, which can be parameterized from a very limited number of experiments and then be used as a so-called soft sensor for predicting granule moisture. It utilizes available process measurements for the estimation of the granule moisture. The development of the model as well as parameter identification and validation experiments are provided. The proposed model paves the way for the application of sophisticated observer concepts. Possible future activities on that topic are outlined in the paper.

## 1. Introduction

In continuous “from powder to tablet” pharmaceutical wet granulation lines, an intermediate drying step is needed to ensure appropriate granule moisture [1,2]. The granule moisture content (further denoted by ‘loss on drying’ or LOD) after the drying step is typically considered an intermediate critical quality attribute of the final drug product [3]. Depending on the type of dryer and its operating mode, the variability of material attributes and process disturbances can lead to varying LOD after drying. Such variations can be mitigated by the implementation of feedback control [4]. However, the realization of feedback control for LOD requires the knowledge of the actual LOD value, which is not easily accessible via real-time measurements.

In common semi-continuous dryers [1,2], multiple separated cells are sequentially filled and used for drying the granules in each cell, such systems have, for example, been implemented by GEA, Bohle or Glatt. In order to measure the LOD in the individual cells, multiple sensors, such as near infrared (NIR) probes or microwave resonance technology (MRT) sensors [5], would be needed. These types of sensors are typically expensive, and therefore, their installation in all dryer cells is not feasible. However, in most systems, standard process sensors capturing the air and material temperatures, the air volume flow and pressure, and the air humidity are implemented. A recent study has used this information to estimate the granule LOD from energy and mass balances [3]. Although a technique is introduced to approximately model dynamic changes of the dryer parameters, e.g., dryer rotation speed or inlet material mass flow, a more rigorous mechanistic modeling approach would be needed to predict the impact of these dynamic parameter changes accurately. The potential of applying a process model for optimizing the startup procedure of pharmaceutical drying units is demonstrated in [6,7]. The process model used in these studies is not based on first principles but on statistically obtained factor–response relationships. This approach offers the advantage of using simple models but has the drawback of needing an extensive experimental effort to obtain the model parameters.

Models that are based on a mechanistic understanding of the process can reduce this experimental effort. Therefore, our study aimed at providing a process model, which is based on a mechanistic understanding of the drying process. Nevertheless, any underlying process model (PM) should be kept as simple as possible for two reasons: First, the computational effort for performing simulations should be kept low, allowing the execution of the simulation in parallel with the real process, as required for model-based control strategies. A real-time execution of the PM is then considered a soft sensor for predicting LOD. Specifically, measured process inputs are the input for the PM, which computes estimates of the LOD. Second, the simple structure of the model should allow the development of observer concepts [8,9] in the future. Such concepts use the measured system inputs and system outputs, as well as a mathematical model of the system to compute an estimate of non-measured system states.

The present work outlines the development of such a PM, which is then used as a straightforward soft sensor. The proposed soft sensor uses available process data of an industrial GEA ConsiGma^TM^-25 powder-to-tablet wet granulation line in order to predict the LOD of the granules in the dryer.

The next section introduces the plant setup, the available process data acquisition, and details on the proposed soft sensor approach and the parametrization. Validation results of the soft sensor approach are provided in Section 3. Section 4 concludes the paper and gives an outlook on future activities.

## 2. Materials and Methods

### 2.1. Process Setup and Problem Statement

**Figure 1:**
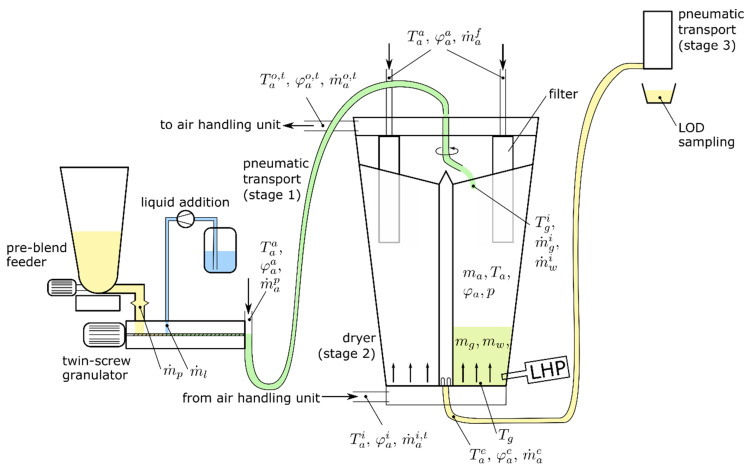
Schematic of the process setup including the process variables. A loss in weight feeder and a liquid pump are used for feeding raw materials into the twin-screw granulator. After granulation, the wet granules are transported to the dryer via pneumatic transport (stage 1). After the drying (stage 2) has been finished in the dryer, the dried material is transported to the downstream unit operations via another pneumatic transport (stage 3). See the nomenclature section for a variable description.

A schematic of the process units—feeding, wet granulation, and drying—is shown in Figure 1. A pre-blend and a granulation liquid are fed into a twin-screw wet granulator and the produced wet granules are then transported to the fluid bed dryer by a pneumatic transport system.

The dryer used in the ConsiGma^TM^-25 line (GEA Pharma Systems, Wommelgem, Belgium) consists of 6 cells, which are sequentially filled with wet granules (stage 1). An air handling unit (AHU) provides a defined air volume flow at a controlled temperature to the dryer and the granules are dried (stage 2). After the drying process in one cell is finished, the respective cell is emptied pneumatically (stage 3). Depending on the required drying time, a certain number of cells is filled with material at a time.

During stage 1, a constant air mass flow from the granulator to the dryer, ${\dot{m}}_{a}^{p}$, is used to transport the granules into the dryer. Every 180 s, the outlet of this transport line is switched from one cell to the next cell. All air masses and air mass flow rates refer to dry air. The mass / mass flow rate of humid air is obtained by multiplication with $\left(1+x_{a}\right)$, where $x_{a}$ is the corresponding water content in kg of water per kg of dry air. The air temperature and relative humidity of the ambient air are denoted by $T_{a}^{a}$ and $\varphi_{a}^{a}$, respectively. Subscripts are used for indicating the material, e.g., air, superscripts denote the location, e.g., ambient. A “*t*” in the superscript refers to the total mass flow, in contrast to individual mass flows of specific cells. Air pressure is denoted by p. The dry mass of granules in the dryer is denoted by $m_{g}$, the water mass in the granules is given by $m_{w}$. The drying—taking place during stage 2—is accomplished by means of inlet air coming from the air handling unit (total inlet air mass flow ${\dot{m}}_{a}^{i,t}$, inlet air temperature $T_{a}^{i}$, and inlet air humidity $\varphi_{a}^{i}$). The air properties at the outlet of the dryer are denoted by superscript “o”. During emptying in stage 3, which takes 30 s, an intermittent air flow (denoted ${\dot{m}}_{a}^{f})$ is injected into the respective filter to clean the filter from dust via blow-back. This intermittent air flow is active for 10 s in a pulse-wise fashion during the emptying phase. The emptying itself is achieved by a pneumatic transport system. During the emptying phase, part of the inlet air mass flow ${\dot{m}}_{a}^{i,t}$ and part of the filter blow-back air mass flow ${\dot{m}}_{a}^{f}$ exits the dryer as emptying air mass flow ${\dot{m}}_{a}^{e}$. In standard operation mode, the drying time of all cells is fixed at a constant time interval. Changes in the inlet material properties and process parameters, such as the initial LOD ($w_{p}$) of the pre-blend material, liquid to solid ratio at the granulation unit, granule mass flow rate (composed of dry granule mass flow ${\dot{m}}_{g}^{i}$ and mass flow of water contained in the granules ${\dot{m}}_{w}^{i}$), dryer inlet air temperature $T_{a}^{i}$ and humidity $\varphi_{a}^{i}$, and dryer inlet air mass flow ${\dot{m}}_{a}^{i,t}$, are influencing the LOD $(w_{g}$) of the granules.

**Figure 2:**
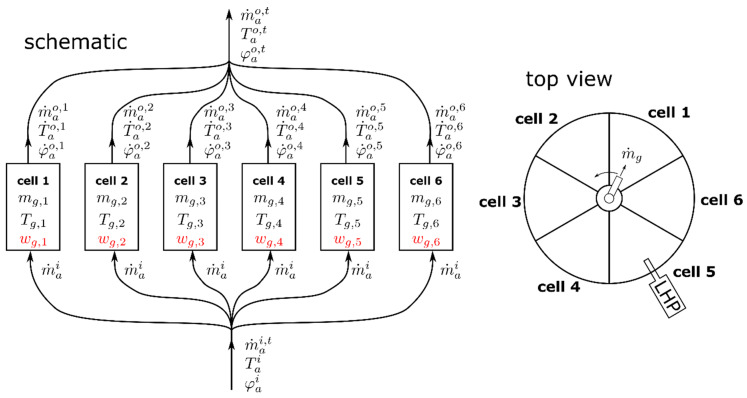
Sketch of the dryer cells and the airflow distribution. Airflows needed for granule transport and for filter blowback are omitted for the sake of simplicity. Dry mass of granules and the corresponding water content (wet basis) in cell k are denoted by $m_{g,k}$ and $w_{g,k}$, respectively.

Cell number 5 of the dryer is equipped with a Lighthouse Probe^TM^ by GEA (LHP) that enables the use of an NIR spectrometer (NDC FP710e, NDC Infrared Engineering, Maldon, Essex, United Kingdom). The measurement signal is indicating the LOD of the granules in cell 5. In all of the six cells, a PT100 temperature sensor (JUMO PT 100—type 902044, JUMO GmbH & Co. KG, Fulda, Germany) is installed, which captures the material/air temperature close to the bottom of the dryer. Note that the granules form a shallow fluidized bed and therefore, it is a valid assumption that the temperature in the bed is uniform. Furthermore, the inlet and outlet air temperature ($T_{a}^{i},\;T_{a}^{o}$) and humidity ($\varphi_{a}^{i},\;\varphi_{a}^{o}$), as well as the total inlet air volume flow rate ${\dot{V}}_{a}^{i,t}$, are measured. A sketch of the dryer is shown in Figure 2. As indicated in that figure, the air condition ($T_{a}^{o,t},\;\varphi_{a}^{o,t}$) of the total dryer outlet air, which is a mixture of the individual cell’s outlet air, is measured. However, no information on the individual cell outlet air temperature or humidity is available.

Although the cells are geometrically identical, there might be cell-to-cell variation in terms of the air volume flow and air temperature, because the inlet air is entering radially—in a non-symmetric manner—from one side of the dryer. This construction could ultimately induce different air volume flows and temperatures in the single cells. However, the temperature differences between the cells measured in the empty dryer at the nominal air flow were in the range of only a few degrees Celsius. Therefore, for the paper at hand, these effects were neglected (see the assumption in Section 2.3.1).

The proposed soft sensor should be capable of estimating the LOD of the granules in the respective dryer cell k, i.e., $w_{g,k}=\frac{m_{w,k}}{m_{g,k}+m_{w,k}\;}\times 100\%$ from the available process data. Possible applications of the developed soft sensor for the estimation of the individual cells’ LOD could be to establish a redundancy to the LHP or even act as a low budget replacement of the LHP. The availability of the LOD information could further be used for improving the operating performance of the dryer, see Section 4. In the remainder of the paper, the cell index k is omitted, because the presented equations are valid for each of the cells.

For model parametrization and validation of the soft sensor approach, experiments were conducted. The model formulation given in Table 1 was used.

### 2.2. Process Data Acquisition

**Figure 3:**
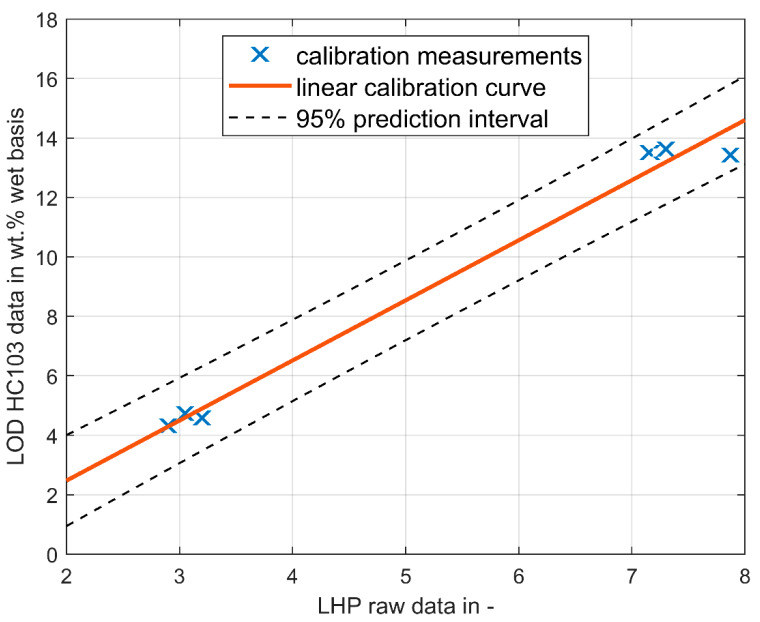
Calibration curve Lighthouse Probe^TM^ (LHP).

Two sets of experiments were executed. The first set was used to identify the model parameters, the second set provides validation data. The process data were acquired via SIPAT [10], which is connected to the SCADA system of the ConsiGma^TM^ line via OPC DA. All the data were captured “as is”, except for the LHP readings on the granule LOD, where a calibration of the LOD data was done. Granules at different LOD were produced and presented to the LHP in a suitable small bucket. Subsequently, the LOD was determined by means of an LOD scale (Mettler Toledo Moisture Analyzer HC103, Mettler-Toldeo GmbH, Vienna, Austria), which was configured by a switch-off criterion of 1 mg/90 s. The values indicated in Figure 3 were measured and the calibration curve:

(1) $$LOD_{LHP}=2.0218\times LOD_{LHP,raw}-1.571\;\left[\%\right],$$

was fitted, as shown in Figure 3. As suggested in the manual of the ConsiGma^TM^ line [11], a linear calibration curve was used. The linearity of the calibration curve was validated in internal studies in the past, and therefore, in the current paper, the two extreme values (measured in triplicate) were assumed to be sufficient information for defining the calibration curve.

### 2.3. Soft Sensor

The proposed soft sensor is based on a dynamic model of the dryer. First, a mathematical model of one dryer cell is developed, which is based on energy and mass balances. Then, six of those models are interconnected in order to model the ConsiGma^TM^ dryer.

#### 2.3.1. Model of One Dryer Cell

The following assumptions and simplifications are made for modelling one dryer cell:

- The overall air flow entering the dryer is equally distributed between the 6 cells, independent of their granule fill level, i.e.,:
  (2) $${\dot{m}}_{a}^{i}=\frac{{\dot{m}}_{a}^{i,t}}{6}.$$
- The total inlet air mass flow ${\dot{m}}_{a}^{i,t}$ of dry air is obtained from the measured volume flow of air ${\dot{V}}_{a}^{meas}$ and the measured inlet air temperature, humidity, and pressure. The computation of the mass balance of the dryer from the captured process data revealed minor inconsistencies between the measured air humidity gain (from dryer inlet to dryer outlet) and the expected humidity gain computed from the inlet air humidity and the granule water content decrease. They are likely linked to measurement uncertainties of the volume flow meter. For their compensation, a correction factor $k_{V}$ is introduced in the computation of the total air volume flow ${\dot{V}}_{a}^{i,t}$ from its measured value, i.e.,:
  (3) $${\dot{V}}_{a}^{i,t}=k_{V}{\dot{V}}_{a}^{meas}.$$
  This correction factor is considered as a parameter to be identified, see Section 2.3.2. From the inlet air flow rate ${\dot{V}}_{a}^{i,t}$, the inlet air water content $x_{a}^{i}$, and the partial water pressure $p_{v}^{i}$ (which are computed via Equations (15)–(17)), the inlet air mass flow of dry air is computed with the help of the specific gas constant of water vapor $R_{v}$ via [3]:
  (4) $${\dot{m}}_{a}^{i,t}=\frac{p_{v}^{i}\;{\dot{V}}_{a}^{i,t}}{x_{a}^{i}\;R_{v}\;(T_{a}^{i}+273.15\;K)\;}.$$
- During emptying of the dryer cell, a constant portion $\alpha_{in,e}$ of the inlet air mass flow exits the dryer via the pneumatic transport system used for emptying. Furthermore, it is assumed that part $\alpha_{f,e}$ of the blowback air exits the dryer via the same route, i.e.,:
  (5) $${\dot{m}}_{a}^{e}=\alpha_{in,e}{\dot{m}}_{a}^{i,t}+\alpha_{f,e}{\dot{m}}_{a}^{f}.$$
  The values of $\alpha_{in,e}=1/35$, $\alpha_{f,e}=0.13$ and filter blowback volume flow ${\dot{V}}_{a}^{f}=20\;m^{3}/h$ were selected manually during the parameter identification procedure. Filter blow back air mass flow ${\dot{m}}_{a}^{f}$ was computed from ${\dot{V}}_{a}^{f}$ using the ambient air condition.
- For modelling the heat transfer from the air in the dryer to the environment, the heat capacity of the outer dryer wall is neglected. The heat flow is assumed to be proportional to the temperature difference between air in the dryer and ambient air.
- Heat transfer to neighboring cells through the separating walls is neglected.
- Outlet air flows of the individual cells are assumed to be perfectly mixed before they reach the outlet air temperature and humidity sensor.
- The influence of the air temperature $T_{a}$ on the air density and consequently on the air mass $m_{a}$ inside the dryer cells is neglected, i.e., it is assumed that $m_{a}$ is constant. Furthermore, space occupied by the granules that would reduce $m_{a}$ is neglected.
- Air condition (temperature, humidity, and pressure) of the filter blowback air and of the granule transport air are assumed to be the ambient air condition.

**Figure 4:**
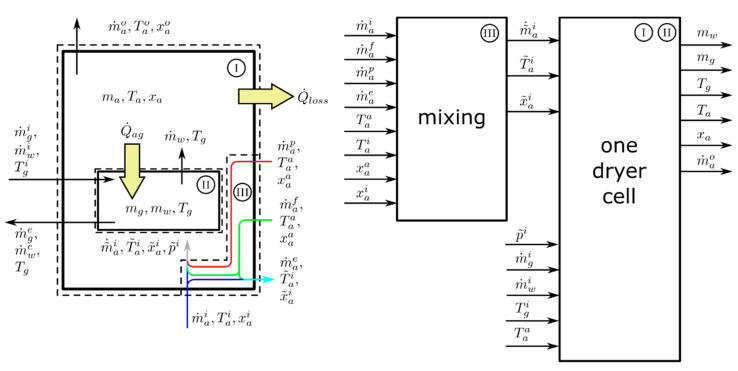
Energy and mass transfer, model inputs, and model outputs. The left part of the figure shows the boundaries of the considered sub-systems, the right part of the figure shows the blocks that were used for model implementation, i.e., the mixing (sub-system III) was implemented as one separate block, whereas air and granule mass and energy balances (sub-systems I and II) are implemented in a separate block.

The inputs and outputs of the model are shown in Figure 4. The implementation of the model was done as shown by the blocks in Figure 4: The mixing of dryer inlet air flow, pneumatic transport air flow, and filter blowback air flow to compute the net inlet air flow and air condition (indicated by a “~”) was implemented in a dedicated mixing block (denoted by III). Information on cell filling and emptying, which is also needed in order to determine all inputs of the mixing block, is derived from line operation data. The sections denoted by I and II are implemented in another dedicated block. Equations (1)–(14) are evaluated in this block. The software Matlab^®^/Simulink^®^, Release 2018b (The MathWorks, Inc., Natick, MA, USA) was used to implement the dryer model.

The mass balances for the granules, the water and the air in (I) and (II), are given by:

(6) $$\frac{d\;m_{g}}{dt}={\dot{m}}_{g}^{i}-{\dot{m}}_{g}^{e},$$

(7) $$\frac{d\;m_{w}}{dt}={\dot{m}}_{w}^{i}-{\dot{m}}_{w}^{e}-{\dot{m}}_{w\;},$$

(8) $$\frac{d\;m_{a}}{dt}=0={\dot{\tilde{m}}}_{a}^{i}-{\dot{m}}_{a}^{o},$$

(9) $$\frac{d\;\left(m_{a}\;x_{a}\right)}{dt}=m_{a}\frac{d\;x_{a}}{dt}={\dot{\tilde{m}}}_{a}^{i}{\tilde{x}}_{a}^{i}-{\dot{m}}_{a}^{o}x_{a}+{\dot{m}}_{w}.$$

Please see Figure 4 for the meaning of the variables used in Equations (6)–(9). The terms $-{\dot{m}}_{g}^{e}$ and $-{\dot{m}}_{w}^{e}$ in Equations (6) and (7) are non-zero during emptying of the dryer. As the emptying is finished within a few seconds, i.e., in a time span much shorter than one drying cycle (see Section 3), $m_{w}$ and $m_{g}$ are set to zero during emptying. This procedure offers the advantage that the actual values of $-{\dot{m}}_{g}^{e}$ and $-{\dot{m}}_{w}^{e}$ are not needed in order to perform the simulation. To obtain the required inputs of the model (I) and (II) from the available process data, ideal mixing and energy conservation was assumed in block (III). The net inlet mass flow of dry air ${\dot{\tilde{m}}}_{a}^{i}$ and its corresponding temperature ${\tilde{T}}_{a}^{i}$ and water content ${\tilde{x}}_{a}^{i}$ are computed under this assumption the energy balances in (I) and (II) are given by:

(10) $$\frac{dU_{gw}}{dt}={\dot{m}}_{g}^{i}c_{g}T_{g}^{i}+{\dot{m}}_{w}^{i}c_{w}T_{g}^{i}-{\dot{m}}_{w}c_{w}T_{g}-{\dot{m}}_{g}^{e}c_{g}T_{g}-{\dot{m}}_{w}^{e}c_{w}T_{g}+{\dot{Q}}_{ag},$$

(11) $$\frac{dU_{av}}{dt}={\dot{\tilde{m}}}_{a}^{i}{\tilde{h}}_{a}^{i}-{\dot{m}}_{a}^{o}h_{a}+{\dot{m}}_{w}c_{w}T_{g}-{\dot{Q}}_{loss}-{\dot{Q}}_{ag}={\dot{\tilde{m}}}_{a}^{i}\left({\tilde{h}}_{a}^{i}-h_{a}\right)+{\dot{m}}_{w}c_{w}T_{g}-{\dot{Q}}_{loss}-{\dot{Q}}_{ag}.$$

For the terms $-{\dot{m}}_{g}^{e}c_{g}T_{g}$ and $-{\dot{m}}_{w}^{e}c_{w}T_{g}$ in Equation (10), the same argumentation as given in the paragraph above was applied. Consequently, during emptying, the internal energy of granules $U_{gw}$ was initialized to zero. During drying, it is assumed that the water contained in the granules is transferred to the air, where it immediately evaporates. The internal energy of air and granules is computed according to:

(12) $$U_{gw}=m_{g}c_{g}T_{g}+m_{w}c_{w}T_{g},$$

(13) $$U_{av}=m_{a}(c_{p,a}T_{a}+x_{a}\left(c_{p,v}T_{a}+\Delta h_{e}\right)).$$

Equations (12) and (13) are rearranged to compute $T_{g}$ and $T_{a}$, respectively. The specific enthalpy $h_{a}$ of the humid air, but based on the dry air mass $m_{a}$, is computed by:

(14) $$h_{a}=c_{p,a}T_{a}+x_{a}\;\left(\Delta h_{e}+c_{p,v}\;T_{a}\right).$$

The conversion from relative humidity $\varphi_{a}$ to water content $x_{a}$ and vice versa is done via the relations provided in [12,13]:

(15) $$\varphi_{a}=\frac{p_{v}}{p_{v,s}},$$

(16) $$x_{a}=0.622\frac{p_{v}}{p-p_{v}},$$

(17) $$p_{v,s}=611\times exp\;\left(-1.91275\times 10^{-4}+7.258\times 10^{-2}\times T-2.939\times 10^{-4}\times T^{2}+9.841\times 10^{-7}\cdot T^{3}-1.92\times 10^{-9}\times T^{4}\right).$$

Here, the conversion factor of 0.622 refers to the conversion of molar fractions to mass fractions. The pressures p, $p_{v}$, and $p_{v,s}$ denote the total pressure, the partial pressure of water vapor, and the partial pressure of saturated water vapor, respectively. The outlet air condition of the dryer in terms of temperature and humidity is obtained from the outlet air conditions of the individual cells via:

(18) $${\dot{m}}_{a}^{o,t}=\sum_{k=1}^{6}{\dot{m}}_{a}^{o,k},$$

(19) $$x_{a}^{o,t}=\frac{1}{{\dot{m}}_{a}^{o,t}}\sum_{k=1}^{6}x_{a}^{o,k}\;{\dot{m}}_{a}^{o,k},$$

(20) $$T_{a}^{o,t}=\frac{\sum_{k=1}^{6}{\dot{m}}_{a}^{o,k}\;h_{a,k}-x_{a}^{o,t}\;\Delta h_{e}\;{\dot{m}}_{a}^{o,t}}{{\dot{m}}_{a}^{o,t}\;(c_{p,a}+x_{a}^{o,t}c_{p,v})}.$$

The terms describing energy and mass transfer, ${\dot{Q}}_{ag}$, ${\dot{Q}}_{loss}$, and ${\dot{m}}_{w}$, respectively, are approximated by:

(21) $${\dot{Q}}_{ag}=\left[\left(\left(1-k_{1}\right){\tilde{T}}_{a}^{i}+k_{1}T_{a}\right)-T_{g}\right]k_{ag},$$

(22) $${\dot{Q}}_{loss}=\left[\left(\left(1-k_{1}\right){\tilde{T}}_{a}^{i}+k_{1}T_{a}\right)-T_{a}^{a}\right]k_{loss},$$

(23) $${\dot{m}}_{w}=k_{m}\;m_{w}\left(p_{v,s}-p_{v}\right){\dot{\tilde{m}}}_{a}^{i}.$$

In Equation (23), the computation of the partial saturation pressure of water vapor $p_{v,s}$ and the partial pressure of water vapor $p_{v}$ is based on the inlet air-temperature, -humidity, and -pressure. By this simplified computation of the water transfer from the granules to the drying air, the outlet air of the individual cells takes relative humidity values above 100% for some operating conditions. After mixing the individual air streams of the six cells, the mixed air has a relative humidity below 100% for all the investigated operating conditions. For the sake of model simplicity, which is beneficial for the intended application to the observer design, and due to the sufficient prediction quality of the measured outputs (see Section 3.2), this behavior of the model was accepted.

In the literature [14,15], more detailed models on the energy and mass transfer are given, which might improve the model accuracy. However, as the prediction performance of the presented model was satisfactory, and in order to keep the model simple, the strongly simplified phenomenological approach given by Equations (21)–(23) was chosen.

#### 2.3.2. Parametrization of the Dryer Model

For model parametrization, the dryer model is fed with experimental data obtained from the ConsiGma^TM^-25 line. The identification of the model parameters $k_{m}$, $k_{ag}$, $k_{loss}$, $k_{1}$, $m_{a}$, $k_{V}$, and $k_{sep}$ is accomplished by solving the following optimization problem:

(24) $$F_{1}=\sum_{t=t_{start}}^{t_{end}}{\left[0.25\;(T_{a}^{o,t}-{\hat{T}}_{a}^{o,t})\right]}^{2},$$

(25) $$F_{2}=\sum_{t=t_{start}}^{t_{end}}{\left[1000\;\left(x_{a}^{o,t}-{\hat{x}}_{a}^{o,t}\right)\right]}^{2},$$

(26) $$F_{3}=\sum_{t=t_{start}}^{t_{end}}\sum_{k=1}^{6}{\left[T_{g,k}-(k_{sep}{\tilde{T}}_{a}^{i}+\left(1-k_{sep}\right){\hat{T}}_{g,k}\right]}^{2},$$

(27) $$F_{4}=\sum_{t\in “drying”}^{}{\left[2\;(w_{g,5}-{\hat{w}}_{g,5})\right]}^{2},$$

(28) $$\underset{k_{m},\;k_{ag},\;k_{loss},k_{1},m_{a},k_{V},k_{sep}}{\min}\underset{i}{\max}F_{i}.$$

In Equations (24)–(27), the signals denoted by a hat indicate the model output, the other signals show the measurements. The weighting factors of 0.25, 1000, 1, and 2 for the individual terms were selected empirically. Their choice is based on the idea that (i) the error of the outlet air water content should be in the same numerical range as the temperature errors, (ii) that the outlet air temperature error is weighted less compared to the individual cell temperature errors, and (iii) that the LOD error is weighted stronger, since it is the quantity of primary interest. Via Equation (28), the maximum value of $F_{1}$ to $F_{4}$ should be minimized by adjusting the model parameters.

**Figure 5:**
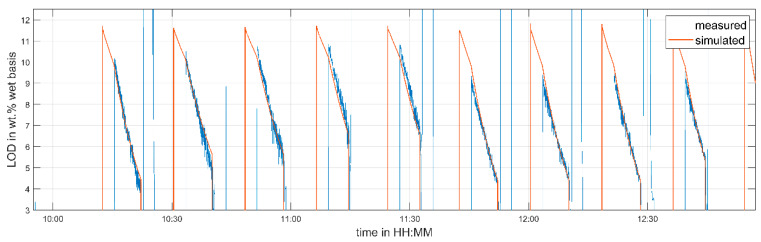
Measured and predicted LOD in cell 5—parameter identification run.

The considered time interval defined by $t_{start}$ and $t_{end}$ was selected based on the available identification data, see Section 3.1. The interval denoted by “drying” refers to the time, where LOD measurements of cell number 5 are available, see Figure 5. The convex combination of ${\tilde{T}}_{a}^{i}$ and ${\hat{T}}_{g,k}$ via $k_{sep}$, which is introduced in Equation (26), is needed, because the temperature sensors installed in the individual cells do not solely measure granule temperature, but a combination of the air temperature and granule temperature. The value of $k_{sep}$ was obtained during parameter identification. Furthermore, the simulated granule temperatures were filtered by a first-order low pass filter with a time constant of 30 s, approximating the temperature sensor dynamics. The identified parameters are summarized in Table 2.

#### 2.3.3. Application of the Model as a Soft Sensor—Trivial Observer

A so-called trivial observer can be constructed by using a one-to-one copy of the system model proposed above. Solely, the information on the inlet air temperature, humidity, and air volume flow, as well as the information on the granule properties at the dryer inlet are used for predicting the LOD. In addition, the plant model is also capable of predicting the individual cell temperatures $T_{g,k}$. The application of this type of soft sensor is demonstrated in Section 3.2.

## 3. Results

### 3.1. Parametrization Experiments

To parameterize the soft sensor, experiments at different process conditions were conducted. The drying time, inlet air temperature, and inlet air volume flow were varied. Table 3 summarizes the selected process settings of the identification run. The time interval from 10:27–12:56 was used for parameter identification. The time from 10:02–10:27 was ignored, because this interval was considered as the heat-up phase of the granulation line.

**Figure 6:**
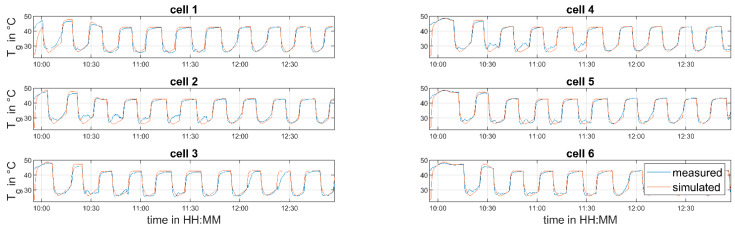
Measured and predicted granule temperatures—parameter identification run.

A comparison of the measured and predicted LOD values and granule temperatures is given in Figure 5 and Figure 6, respectively. The predicted LOD matches the measured one quite well, after the start-up phase of the line (10:42) is completed. The prediction of the granule temperatures shows a good agreement after 11:40. The fluctuations in the measured temperatures before that time are caused by material sticking to the sensors due to non-optimal fluidization. As this effect is unmodeled, the deviations between measurement and simulation are explainable.

### 3.2. Validation Experiments

In order to validate the created process model, another set of experiments was performed as shown in Table 4.

**Figure 7:**
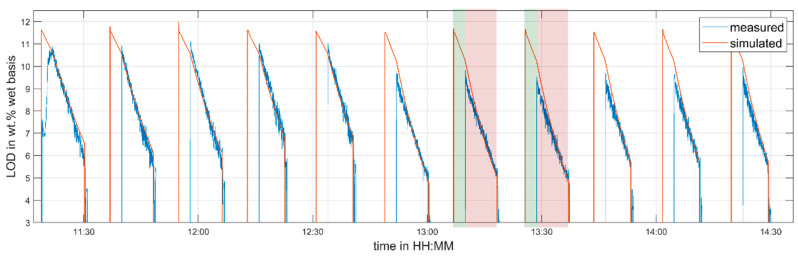
Measured and predicted LOD in cell 5—validation experiment. Two drying cycles are highlighted (filling in green, drying in red).

**Figure 8:**
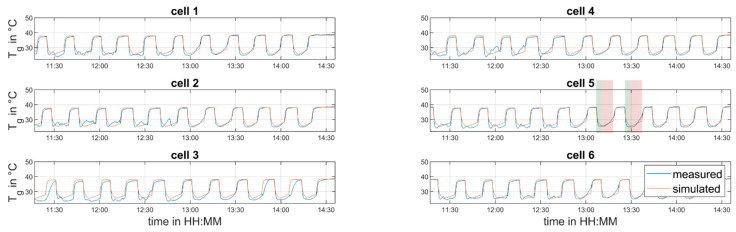
Measured and predicted granule temperatures—validation experiment. Two drying cycles are highlighted (filling in green, drying in red).

Figure 7 and Figure 8 show the comparison between predicted and measured data. As it was done during the parametrization run, the process inlet conditions were varied throughout the experiment. Nevertheless, the validation dataset differs from the parametrization dataset in terms of the inlet air temperature. A different inlet air temperature was used to demonstrate the applicability of the proposed model to adapt to different process settings. As in the parameter identification dataset, the LOD is predicted well after the startup phase of the line (11:45). The temperature fluctuations in the granule temperature measurement visible in Figure 8 can again be explained by non-perfect fluidization for lower air flow rates. However, at the air volume flow of $400{\;m}^{3}/h$, the agreement of the measured and predicted temperature is quite good.

**Figure 9:**
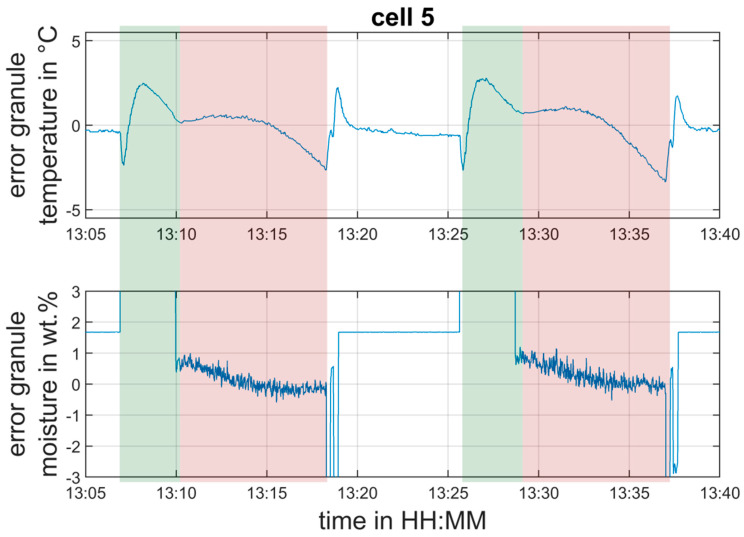
Detailed view of the two drying cycles highlighted in Figure 7 and Figure 8. Estimation error of granule moisture and temperature in cell 5—validation experiment.

Figure 9 shows a more detailed view of the estimation errors $e_{w,5}={\hat{w}}_{g,5}-w_{g,5}$ and $e_{T_{g,5}}=(k_{sep}{\tilde{T}}_{a}^{i}+\left(1-k_{sep}\right){\hat{T}}_{g,k})-T_{g,5}$ (LOD and granule temperature in cell 5) in the time interval between 13:05 and 13:40. The estimation error for LOD is between 0% and 1%, and for the granule temperature, it is in the range of ±2.5 °C during drying. The short-term increase of the error during filling of the cell is not a big issue for the concept presented in the paper, because the main interest is the estimation of the mentioned quantities during the actual drying phase, which starts after the chamber is totally filled.

A simulation scenario that covers a time of 3 h was executed on an Intel XEON E3-1245V2, 3.4 Ghz with 16 GB of memory workstation. The execution took approximately 25 s, i.e., 0.23% of the simulation scenario time. Therefore, the model is—in contrast to most CFD or DEM simulation models—well suited for real-time execution.

## 4. Discussion and Conclusions

**Figure 10:**
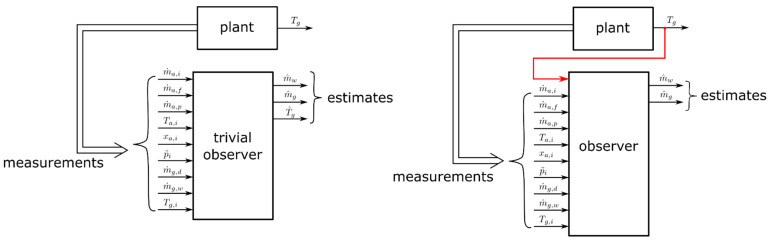
Trivial observer (**left**), state observer (**right**).

The ability of predicting the granule moisture in the ConsiGma 25^TM^ dryer was successfully demonstrated by the proposed dynamic process model. Its performance—when being used as a soft sensor in the form of a so-called trivial observer (i.e., only plant inputs are used for predicting state variables, see Figure 10, left)—reveals its applicability for monitoring the LOD in the individual dryer cells.

Furthermore, it was shown that the temperature of the granules in the cells could be predicted reasonably well, too. One might not be interested in predicting that temperature, since it is directly available by measurement. However, as this is the only measurement that is directly related to the state of the granules in the individual cells, the implementation of observers using this information for improving LOD prediction seems promising. Such observer concepts utilize the available measured outputs in order to improve the estimation of unknown states [9,16,17]. For the investigated dryer, the output would be the individual cell temperature, while the LOD is computed from the state variables $m_{w}$ and $m_{g}$ to be estimated, see Figure 10, right. Future work will focus on the development of such observers.

The obtained LOD values could further be used as an LOD-based drying end point detection, i.e., the drying can be stopped at the predicted granule moisture instead of using a constant drying time. This approach could significantly reduce the variability in the batch to batch granule quality. It is well known that in dryers, a certain variability exists, since the airflow in the chamber likely is not always identical between cells. Thus, end-point detection could eliminate the variability.

Approaches exist that use available process data for drying end-point detection. However, these approaches often use indirect parameters (e.g., temperature increase during drying) [18] as a criterion for drying end-point detection. With the proposed soft sensor, the drying end point can be directly specified by the desired LOD value, which is advantageous compared to approaches based on indirect parameters [19].

The performance gain of a fluid bed dryer via implementation of feedback control (i.e., adjustment of process parameters like air volume flow, inlet air temperature) was demonstrated by [4,20]. The successful implementation of the observer concepts mentioned in the paragraph above would pave the way to develop feedback control based on the estimated LOD values for this type of six-segmented dryer.

## Figures and Tables

**Table 1:** Used model formulation.

Material	wt. %
Lactose (GranuLac^®^ 200, MEGGLE, Wasserburg am Inn, Germany)	72
Corn starch (Maisita, Native Maisstärke, Agrana, Vienna, Austria)	24
Povidon PVP (Kollidon^®^ 90 F, BASF/BTC Europe GmbH, Monheim am Rhein, Germany)	4

**Table 2:** Model parameters.

Parameter	Value
$k_{1}$	0.381
$k_{ag}$	3.22 J/(K·s)
$k_{loss}$	0.353 J/(K·s)
$k_{m}$	1.77 × 10^−5^ 1/kg
$m_{a}$	0.197 kg
$k_{V}$	0.807
$k_{sep}$	0.1

**Table 3:** Parameter identification—experiments. Bold values indicate changes in process conditions. Note the different units compared to the nomenclature. They were used in this table for better readability of the numbers.

Time	${\dot{m}}_{p}$	${\dot{m}}_{l}\;$	$T_{a,in}$	${\dot{V}}_{a}^{meas}$	$t_{dry}$
hh:mm	kg/h	g/min	°C	$m^{3}/h$	s
10:02–10:20	20	40	50	300	680
10:20–10:28	20	40	50	300	580
10:28–11:00	20	40	45	300	580
11:00–11:39	20	40	45	300	480
11:39–12:33	20	40	45	400	580
12:33–12:56	20	40	45	400	480

**Table 4:** Validation—experiments. Bold values indicate changes in process conditions. Note the different units compared to the nomenclature. They were used in this table for better readability of the numbers.

Time	${\dot{m}}_{p}$	${\dot{m}}_{l}\;$	$T_{a}^{i}$	${\dot{V}}_{a}^{meas}$	$t_{dry}$
hh:mm	kg/h	g/min	°C	$m^{3}/h$	s
11:06–11:28	20	40	40	260	680
11:28–12:06	20	40	40	300	680
12:06–12:46	20	40	40	300	580
12:46–13:40	20	40	40	400	680
13:40–14:20	20	40	40	400	580

## Nomenclature

$c_{p,a}$	J/(kg·K)	specific heat capacity of dry air: $c_{p,a}=1005\;J/\left(\mathrm{kg}\cdot K\right)$ [12] (p. 289)
$c_{p,v}$	J/(kg·K)	specific heat capacity of water vapor: $c_{p,v}=1863\;J/\left(\mathrm{kg}\cdot K\right)$ [12] (p. 289)
$c_{w}$	J/(kg·K)	specific heat capacity of water: $c_{w}=4191\;J/\left(\mathrm{kg}\cdot K\right)$ [12] (p. 289)
$c_{g}$	J/(kg·K)	specific heat capacity of granules (computed from specific heat of lactose [21] (p. 20), corn starch [22] (p. 10) and Povidon PVP [23] (p. 120)): $c_{g}=1321\;J/\left(\mathrm{kg}\cdot K\right)$
$\Delta h_{e}$	J/kg	evaporation enthalpy of water at T=0 °C: $\Delta h_{e}=2501\times 10^{3}\;J/\mathrm{kg}$ [12] (p. 289)
$k_{1}$	-	factor used to obtain “mean” air temperature across cell height
$k_{ag}$	J/(K·s)	proportional factor describing heat transfer from air to granules
$k_{m}$	1/kg	proportional factor describing evaporation rate
$k_{loss}$	J/(K·s)	proportional factor describing heat transfer from air to ambient
$k_{sep}$	-	factor used to obtain cell temperature sensor value from ${\tilde{T}}_{a}^{i}$ and ${\hat{T}}_{g,k}$
$m_{a}$	kg	dry air mass in one dryer cell
$m_{g,k}$	kg	dry granule mass in dryer cell k
$m_{w,k}$	kg	water mass in granules in dryer cell k
${\dot{m}}_{a}^{e}$	kg/s	dryer emptying air mass flow
${\dot{m}}_{a}^{i}$	kg/s	dryer inlet mass flow of dry air into one dryer cell coming from AHU
${\dot{\tilde{m}}}_{a}^{i}$	kg/s	net inlet mass flow of dry air into one dryer cell
${\dot{m}}_{a}^{i,t}$	kg/s	total dryer inlet mass flow of dry air coming from AHU
${\dot{m}}_{a}^{f}$	kg/s	mass flow of dry air for filter blowback
${\dot{m}}_{a}^{o,t}$	kg/s	total dryer outlet mass flow of dry air
${\dot{m}}_{a}^{o,k}$	kg/s	dryer cell k outlet mass flow of dry air
${\dot{m}}_{a}^{p}$	kg/s	mass flow of dry air for granule transport
${\dot{m}}_{p}$	kg/s	mass flow of pre-blend
${\dot{m}}_{g}^{i}$	kg/s	mass flow of dry granules during filling
${\dot{m}}_{w}^{i}$	kg/s	mass flow of water (in wet granules) during filling
${\dot{m}}_{g}^{e}$	kg/s	mass flow of dry granules during emptying
${\dot{m}}_{w}^{e}$	kg/s	mass flow of water (in wet granules) during emptying
${\dot{m}}_{l}$	kg/s	mass flow of granulation liquid
${\dot{m}}_{w}$	kg/s	mass flow of water from granules to air
p	Pa	air pressure
${\tilde{p}}_{}^{i}$	Pa	air pressure of inlet air into one dryer cell
$p_{v}$	Pa	partial pressure of water vapor
$p_{v,s}$	Pa	partial saturation pressure of water vapor
${\dot{Q}}_{loss}$	J/s	heat flow between dryer and surroundings (heat loss)
${\dot{Q}}_{ag}$	J/s	heat exchange between air and granules
$R_{v}$	J/(kg·K)	specific gas constant of water vapor: $R_{v}=461.53\;J/\left(\mathrm{kg}\cdot K\right)$ [12] (p. 286)
$\varphi_{a}$	%	relative humidity of air
$\varphi_{a}^{a}$	%	relative humidity of ambient air
$\varphi_{a}^{e}$	%	relative humidity of air while emptying
$\varphi_{a}^{i}$	%	relative humidity of air coming from the AHU
$\varphi_{a}^{o,t}$	%	relative humidity of total dryer outlet air
$\varphi_{a}^{o,k}$	%	relative humidity of cell k outlet air
$T_{g,k}$	°C	temperature in one dryer cell k
$T_{a}$	°C	air temperature
$T_{a}^{a}$	°C	ambient air temperature
$T_{a}^{e}$	°C	air temperature emptying
$T_{a}^{i}$	°C	air temperature of air coming from AHU
${\tilde{T}}_{a}^{i}$	°C	air temperature of net air entering one dryer cell
$T_{a}^{o,k}$	°C	air temperature outlet of cell k
$T_{a}^{o,t}$	°C	air temperature dryer outlet
$T_{g}^{}$	°C	measured granule temperature
$T_{g}^{i}$	°C	granule temperature of granules being filled
$U_{av}$	J	internal energy of humid air
$U_{gw}$	J	internal energy of wet granules
$w_{g,k}$	%	LOD of granules (wet basis) in dryer cell k ($w_{g,k}=\frac{m_{w,k}}{m_{w,k}+m_{g,k}}\times 100\;\%$)
$w_{p}$	%	initial LOD of pre-blend (wet basis)
$x_{a}$	kg/kg	water content based on dry air mass, i.e., kg water per kg dry air
$x_{a}^{i}$	kg/kg	water content of air coming from AHU
${\tilde{x}}_{a}^{i}$	kg/kg	water content of net air entering one dryer cell
${\dot{V}}_{a}$	$m^{3}/s$	volume flow of air
